# Heterogenous CD8+ T Cell Maturation and ‘Polarization’ in Acute and Convalescent COVID-19 Patients

**DOI:** 10.3390/v14091906

**Published:** 2022-08-28

**Authors:** Igor V. Kudryavtsev, Natalia A. Arsentieva, Zoia R. Korobova, Dmitry V. Isakov, Artem A. Rubinstein, Oleg K. Batsunov, Irina V. Khamitova, Raisa N. Kuznetsova, Tikhon V. Savin, Tatiana V. Akisheva, Oksana V. Stanevich, Aleksandra A. Lebedeva, Evgeny A. Vorobyov, Snejana V. Vorobyova, Alexander N. Kulikov, Maria A. Sharapova, Dmitrii E. Pevtsov, Areg A. Totolian

**Affiliations:** 1 Institute of Experimental Medicine, Akademika Pavlova 12, 197376 Saint Petersburg, Russia; 2 Medical Faculty, First Saint Petersburg State I. Pavlov Medical University, L’va Tolstogo St. 6-8, 197022 Saint Petersburg, Russia; 3 Laboratory of Immunology, Saint Petersburg Pasteur Institute, Mira 14, 197101 Saint Petersburg, Russia; 4 Smorodintsev Research Institute of Influenza, Prof. Popov St. 15/17, 197376 Saint Petersburg, Russia

**Keywords:** COVID-19, SARS-CoV-2, CD3+CD8+, memory CD8+ T cells, Tc1, Tc2, Tc17, chemokine receptors, IL-27, COVID-19 convalescent, post-COVID-19 syndrome

## Abstract

Background. The adaptive antiviral immune response requires interaction between CD8+ T cells, dendritic cells, and Th1 cells for controlling SARS-CoV-2 infection, but the data regarding the role of CD8+ T cells in the acute phase of COVID-19 and post-COVID-19 syndrome are still limited. Methods.. Peripheral blood samples collected from patients with acute COVID-19 (*n =* 71), convalescent subjects bearing serum SARS-CoV-2 N-protein-specific IgG antibodies (*n =* 51), and healthy volunteers with no detectable antibodies to any SARS-CoV-2 proteins (HC, *n =* 46) were analyzed using 10-color flow cytometry. Results. Patients with acute COVID-19 vs. HC and COVID-19 convalescents showed decreased absolute numbers of CD8+ T cells, whereas the frequency of CM and TEMRA CD8+ T cells in acute COVID-19 vs. HC was elevated. COVID-19 convalescents vs. HC had increased naïve and CM cells, whereas TEMRA cells were decreased compared to HC. Cell-surface CD57 was highly expressed by the majority of CD8+ T cells subsets during acute COVID-19, but convalescents had increased CD57 on ‘naïve’, CM, EM4, and pE1 2–3 months post-symptom onset. CXCR5 expression was altered in acute and convalescent COVID-19 subjects, whereas the frequencies of CXCR3+ and CCR4+ cells were decreased in both patient groups vs. HC. COVID-19 convalescents had increased CCR6-expressing CD8+ T cells. Moreover, CXCR3+CCR6- Tc1 cells were decreased in patients with acute COVID-19 and COVID-19 convalescents, whereas Tc2 and Tc17 levels were increased compared to HC. Finally, IL-27 negatively correlated with the CCR6+ cells in acute COVID-19 patients. Conclusions. We described an abnormal CD8+ T cell profile in COVID-19 convalescents, which resulted in lower frequencies of effector subsets (TEMRA and Tc1), higher senescent state (upregulated CD57 on ‘naïve’ and memory cells), and higher frequencies of CD8+ T cell subsets expressing lung tissue and mucosal tissue homing molecules (Tc2, Tc17, and Tc17.1). Thus, our data indicate that COVID-19 can impact the long-term CD8+ T cell immune response.

## 1. Introduction

Initiation of the type 1 immune response, which requires successful interplay between diverse innate (dendritic cells, primarily plasmacytoid DC (pDCs), and conventional type 1 DCs (cDC1), as well as ILC1 and NK cells) and adaptive (type 1 T helper cells and T follicular helper cells accounting for response regulation, as well as B cells and CD8+ T cells executing effector functions) immune cells [1,2], is necessary for efficient elimination of intracellular pathogens, including the SARS-CoV-2 virus. It is worth mentioning that in acute COVID-19, virtually in all immune cell types, prominent alterations are noted, which are enabled both during effector function initiation and execution in antiviral response [3,4,5].

For instance, regardless of the disease severity, peripheral blood pDC and cDC1 counts were lowered in all COVID-19 patients [6,7], whereas an elevated cDC/pDC ratio might be considered a marker of a severe COVID-19 course [8]. In addition, marked alterations were also observed in the circulating DC phenotype related to the downregulated expression of the MHC class I and II, co-stimulation, and lymphoid tissue homing molecules, as well as cytokine production [9,10,11]. Moreover, it was found that the expression level of CD80, CD86, CCR7, and HLA-DR molecules was lowered on pDC and cDC1 examined in COVID-19 patients vs. healthy volunteers after in vitro stimulation with TLR-3, -7, or -8 ligands [8]. Of note, prolonged decreased functional activity of circulating DCs related to the impaired expression of molecules involved in antigen presentation (e.g., HLA-DR), co-stimulation (e.g., CD86 and PD-L1), and migration (e.g., CCR2, CCR7, and β7 integrin) may be observed, which persists for at least 6 months after the acute phase of SARS-CoV-2 infection [8,12]. A similar crucial role in efficient SARS-CoV-2-derived antigen presentation may be accounted for by diverse genetic factors, which may be referred to as specific gene alleles encoding MHC class I [13,14] and transcription factors, as well as cell migration molecules [15,16], ensuring effector cell pool formation and subsequent migration to the site of inflammation. Altogether, it may suggest impaired activity of the key DC subsets in stimulating Th1- and CD8+ T cell-mediated reactions.

In turn, Th1 cells are responsible for cytotoxic CD8+ T cell proliferation and differentiation in response to intracellular pathogens, as well as regulated production of the cytokines IL-2, IL-12, IL-15, and IL-21 required for differentiation of naïve CD8+ T cells and the formation of effector and memory T cell subsets [1,2]. It should be noted that SARS-CoV-2-specific Th1 cells were found in circulation starting from the early stage after the emergence of COVID-19 symptoms [17], so that such cells were able to recognize antigenic epitopes derived from the three relevant viral proteins such as S-, N-, and M-proteins [18,19]. Moreover, Chen et al. [20] and Chen and Wherry [21] suggested that in COVID-19, a positive role might be played by IFNγ-producing Th1 cells, in which augmented activity could be related to a less severe disease course [20,21]. An optimal environment in peripheral lymphoid organs undergoing profound remodeling in COVID-19 is necessary for efficient interaction between dendritic cells, Th1, and CD8+ T cells. In particular, deceased COVID-19 patients were described to have extended areas of splenic white pulp atrophy, as well as detected foci of lymphocyte death within lymphoid follicles and paracortical areas in lymph nodes [22]. Moreover, an increasing number of apoptotic or necrotic cells was also noted in the lymphoid tissue [23], which might be directly related to a lowered peripheral blood lymphocyte count in COVID-19 patients. In addition, it was also observed that the proportion of phagocytosing histiocytes (macrophages) in the sinuses of hilar lymph nodes was elevated in parallel with a decreased total lymphocyte count in severe COVID-19 [24], which might indirectly point to massive cell death in lymphoid tissues during a severe disease course accompanied by a lethal outcome. Hence, altered functioning of peripheral lymphoid tissues may profoundly affect CD8+ T cell functions in COVID-19, as well as impact the efficacy of developing immune memory. Of note, SARS-CoV-2-specific CD8+ T cells were found in at least 70% of convalescent COVID-19 patients [18], whereas their accelerated generation and egress into circulation during the acute phase of COVID-19 were closely related to mild COVID-19 [25]. Therefore, these data may additionally suggest a crucial role played solely by cytotoxic T cells in controlling the infectious process. Thus, our study aimed to analyze the phenotype of peripheral blood CD8+ T cells in acute-phase, as well as successfully recovered, COVID-19 patients.

## 2. Materials and Methods

### 2.1. Patient Characteristics

For our study, we collected 119 blood samples taken from patients with acute COVID-19 (*n =* 71), convalescents (*n =* 51), and healthy volunteers (*n =* 46). All samples were obtained from April to November 2020. All patients within the COVID-19 cohorts were infected with the original ancestral SARS-CoV-2 Wuhan strain, confirmed via genetic testing. Patients with acute COVID-19 were treated at the COVID-19 specialized Department, the First Saint Petersburg State I. Pavlov Medical University, within the period of May to November 2020. Patient age for this group averaged 60 years old (46; 70). The gender ratio was 25/35.2% males and 46/64.8% females. The diagnosis was based both on clinical presentation (fever, sense of fatigue, muscle and joint pains, cough, and pneumonia confirmed by CT-scans) and qualitative PCR detection of SARS-CoV-2 RNA, according to the COVID-19 Guidelines of the Russian Ministry of Healthcare based on the WHO COVID-19 Clinical management: Living guidance [26]. Out of all 71 COVID-19 patients, 49/69.1% and 22/31.0% were diagnosed with moderate and severe disease courses, respectively, based on the criteria provided by the COVID-19 diagnosis and treatment Guidelines of the Russian Ministry of Health. According to medical records, only 3/4.2% of all patients fully recovered from the infection by the time of discharge, and although 35/49.2% showed positive dynamics in their related general condition as well as CT-scan data, a large group of 29/40.8% was discharged without any significant positive changes in lung tissues. There was a small group of patients with acute COVID-19 (4/5.6%) who unfortunately deceased due to infection complications. Blood samples were collected 7 (4; 12) days post-symptom onset (PSO).

A cohort of convalescent patients included individuals who recovered from COVID-19 within one month prior to sample collection. The gender ratio was 21/41.2% males and 30/58.8% females, with patient age averaging 32 (26; 38) years old. At the time of blood sampling, patients showed no clinical symptoms of infection, having negative results for SARS-CoV-2 RT-PCR detection and sufficient levels of anti-SARS-CoV-2 IgG antibodies. Out of this cohort, 24/47.1% and 20/39.2% subjects had mild and moderate COVID-19 courses, and 7/13.7% recovered from severe COVID-19. Blood samples were collected 73 (62; 95) days PSO.

There were also 46 healthy individuals included (22 males and 24 females), from which peripheral blood samples were collected prior to the COVID-19 pandemic. Because of the high median age of the patients with COVID-19, it was problematic to form a control group with healthy volunteers who lacked comorbidities. Therefore, subjects from the healthy volunteer group had a significantly lower median age of 42 (35; 48) years compared to the patients, which posed a limitation for the current study. Due to the limited number of healthy subjects older than 60 years lacking comorbidities, it was difficult to form an age-matched healthy control group, which was reflected in the age gap between the acute COVID-19 group and the healthy control group

All participants provided signed informed consent. The protocol of the study was approved by the Ethics Committee of the Saint Petersburg Pasteur Institute in full accordance with the Declaration of Helsinki.

### 2.2. Sample Collection

Blood samples were collected before treatment initiation. Five milliliters of peripheral blood were collected from each patient in VACUETTEK3EDTA tubes. Collected peripheral blood samples were immediately processed. For cytokines measurement, cell-free plasma samples were obtained after whole blood centrifugation at 300× *g* for 7 min at + 4 °C, followed by placing it into pure 1.5 mL tubes and subsequent centrifugation at 300× *g* for 15 min at + 4 °C again to sediment residual platelets and other blood cells. Finally, each plasma sample was aliquoted and stored at −80 °C until use. T cell immunophenotyping was performed within a few hours (≤6 h) after blood collection. 

### 2.3. Immunophenotyping of Peripheral Blood CD8+ T Cell Subset Maturation Stages and ‘Polarized’ CD8+ T Cell Subsets in Acute and Convalescent COVID-19

**Figure 1:**
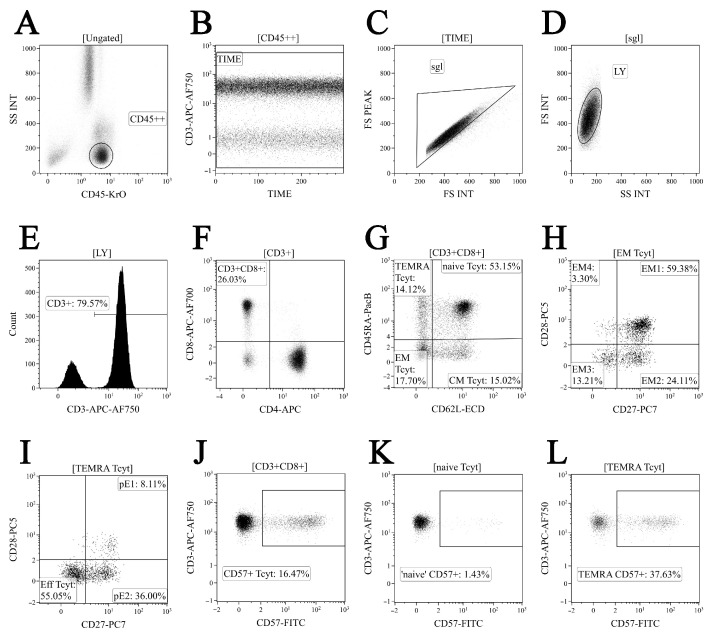
Flow cytometry immunophenotyping gating strategy for CD8+ T cell subset maturation stages and assessing CD57 expression level (shown in dot plots). (**A**) Total lymphocyte subset purification based on side scatter and bright CD45 expression; (**B**) artifact exclusion included time gating; (**C**) doublets exclusion from the analysis by using the ratio between integral and peak forward scatter signals; (**D**) discrimination between lymphocytes and cell debris; (**E**) total CD3 expression-based T cell subset gaiting; (**F**) CD8+ T cells detected within total CD3+ T cell population; (**G**) CD45RA and CD62L co-expression in identifying the four major CD8+ T cell maturation subsets: ‘naïve’ CD45RA+CD62L+ (naïve), central memory CD45RA-CD62L+ (CM), effector memory CD45RA−CD62L− (EM), and terminally differentiated CD45RA-positive effector memory CD45RA+CD62L− (TEMRA) CD8+ T cells; (**H**) EM1 (CD27+CD28+), EM2 (CD27+CD28−), EM3 (CD27−CD28−), and EM4 (CD27−CD28+) subsets were identified within total effector memory CD45RA−CD62L− CD8+ T cells; (**I**) differentiation of terminally differentiated CD8+ T cells (TEMRA) by assessing CD27 and CD28 expression allowed us to subdivide CD8+ T cells into pre-effector type 1 cells (pE1, CD27+CD28+), pre-effector type 2 cells (pE2, CD27+CD28-), and effector cells (E, CD27−CD28−); (**J**–**L**) CD57 expression by total CD8+ T cell population, naïve, and TEMRA CD8+ T cell subsets, respectively.

First, 200 μL of whole peripheral blood samples were stained with the following surface marker-specific fluorochrome-conjugated monoclonal antibodies: Anti-CD57 FITC, anti-CD62L ECD, anti-CD28 PC5.5, anti-CD27 PC7, anti-CD4 APC, anti-CD8 APC-AF700, CD3-APC-AF750, anti-CD45RA Pacific Blue, and anti-CD45 Krome Orange (all antibodies were manufactured by Beckman Coulter, Indianapolis, IN, USA, used according to the manufacturer’s recommendations). Next, all samples were incubated at room temperature in the dark for 10 min followed by red blood cell lysis for 15 min in the dark with 2 mL of the VersaLyse Lysing Solution (Beckman Coulter, Inc., USA) supplied with 50 μL of the IOTest 3 Fixative Solution (Beckman Coulter, Inc., USA). Finally, 200 μL of Flow-Count Fluorospheres (Beckman Coulter, Indianapolis, IN, USA) were added, and sample acquisition was performed using a 3/10 Navios flow cytometer (Beckman Coulter, Indianapolis, IN, USA). At least 20,000 CD8+ T cells were analyzed in each sample. The gating strategy for the major ‘polarized’ CD8+ T cell subset is shown in Figure 1.

**Figure 2:**
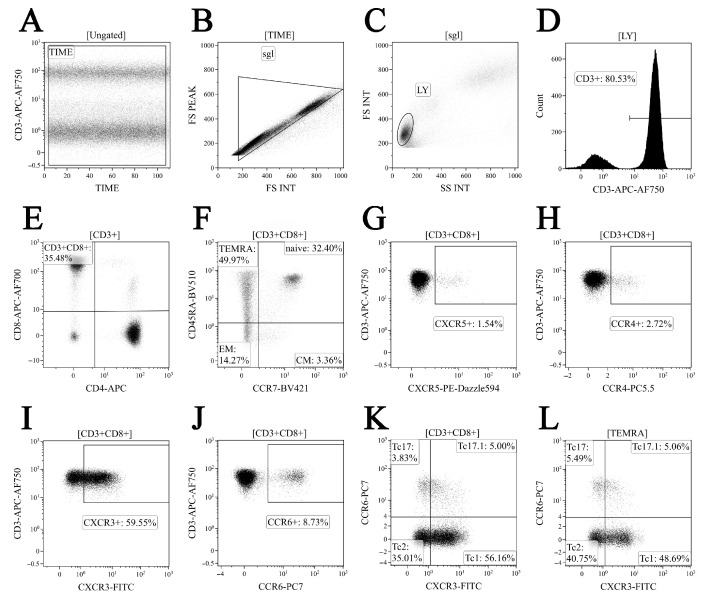
Gating and analysis strategy for ‘polarized’ CD8+ T cell subsets immunophenotyping using flow cytometry. Dot plot (**A**)—artifact exclusion included time gating; dot plot (**B**)—doublets exclusion from the analysis using the ratio between integral and peak forward scatter signals; dot plot (**C**)—total lymphocyte subset purification based on side scatter and forward scatter; dot plot (**D**)—total T cell subset gaiting based on CD3 expression; dot plot (**E**)—detection of CD8+ T cells within total CD3+ T cell subset; dot plot (**F**)—identification of four main CD8+ T cell maturation subsets: ‘naïve’, central memory (CM), effector memory (EM), and TEMRA CD8+ T cells; dot plots (**G**–**L**)—examples of CXCR5, CCR4, CXCR3, and CCR expression by total CD8+ T cells; dot plots—examples of Tc1 (CCR6−CXCR3+), Tc2 (CCR6−CXCR3−), Tc17 (CCR6+CXCR3−), and double-positive Tc17.1 (CCR6+CXCR3+) detection within total CD8+ T cell subset and TEMRA CD8+ T cell, respectively.

Phenotyping of major ‘polarized’ CD8+ T cell subsets was performed using a panel of 10 fluorescently labelled monoclonal antibodies to stain for a specific surface marker panel as described previously [27]. In brief, we used CXCR3-AF488, CXCR5-PE/Dazzle™ 594, CCR4-PerCP/Cy5.5, CCR6-PE/Cy7, CD4-APC, CD8 APC-AF700, CD3-APC/Cy7, CCR7-Brilliant Violet 421, and CD45RA-Brilliant Violet 510 (CD25, CD4, and CD8 were manufactured by Beckman Coulter, Indianapolis, IN, USA, with the remaining other antibodies purchased from BioLegend, Inc., San Diego, CA, USA). First, 200 μL of whole peripheral blood samples were stained according to the manufacturer’s recommendations. Erythrocytes were lysed (15 min in the dark at RT) by adding 2 mL of the VersaLyse Lysing Solution (Beckman Coulter, Inc., USA) supplied with 50 μL of the IOTest 3 Fixative Solution (Beckman Coulter, Inc., USA). Next, cells were washed (7 min, 330 g) twice with a buffer (sterile phosphate-buffered saline (PBS) containing 2% of heat-inactivated fetal bovine serum, Sigma-Aldrich, St. Louis, MO, USA) and resuspended in 0.5 mL PBS containing a 2% neutral buffered formalin solution (Sigma-Aldrich, St. Louis, MO, USA). Sample acquisition was performed using a 3/10 Navios flow cytometer (Beckman Coulter, Indianapolis, IN, USA). At least 20,000 CD8+ T cells were analyzed in each sample. The gating strategy for the major ‘polarized’ CD8+ T cell subsets is shown in Figure 2. 

### 2.4. Serum Cytokine and Chemokine Measurement

Cytokine and chemokine levels were determined using a multiplex analysis performed on the fluorescently labeled magnetic beads with MILLIPLEX^®^ MAP Human Cytokine/Chemokine/Growth Factor Panel (HCYTA-60K-PX48, MilliporeSigma, Burlington, MA, USA), according to the manufacturer’s instructions and protocol using the Luminex MAGPIX instrument system (Luminex, Austin, TX, USA).

### 2.5. Statistical Analysis

The flow cytometry data were analyzed using Kaluza software v2.3 (Beckman Coulter, Indianapolis, IN, USA). Statistical analysis was performed with Statistica 7.0 (StatSoft, Tulsa, OK, USA) and GraphPad Prism 8 (GraphPad software Inc., San Diego, CA, USA) software packages. Normality was checked using Pearson’s chi-squared test. All flow cytometry data were presented as a percentage of positive cells. The absolute number of CD8+ T cell subsets was calculated using a ‘no-wash’ technique and Flow-Count Fluorospheres (Beckman Coulter, Indianapolis, IN, USA), with a suspension of fluorescent latex microbeads used to determine absolute counts on the flow cytometer. All data were presented as the median and interquartile range, Me (Q25;Q75). The inter-group differences were analyzed using a nonparametric Mann–Whitney U-test. The inter-group differences were considered significant with a *p* < 0.05 value. A correlation analysis was performed using the nonparametric Spearman rank test, and significance was set at *p* < 0.05.

## 3. Results

### 3.1. Alterations in Major Peripheral Blood T Cell Subsets in COVID-19 Patients 

**Figure 3:**
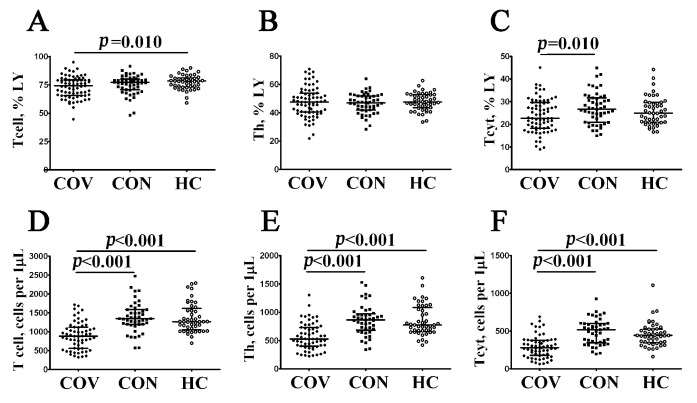
Comparison of relative and absolute frequencies of major T cell subsets in patient with acute COVID-19 and convalescent COVID-19 individuals. Scatter plots (**A**–**C**) and (**D**–**F**) showing the percentages (the percentage of T cell subset within total lymphocyte population) and absolute numbers (number of cells per 1 μL of peripheral blood) of T cells (CD3+), T-helpers (Th, CD3+CD4+), and CD8+ T cells (Tcyt, CD3+CD8+), respectively. Black circles denote patients with acute COVID-19 (COV, *n =* 71); black squares—convalescent COVID-19 individuals (CON, *n =* 51); white circles—healthy control (HC, *n =* 46). Each dot represents individual subject, and horizontal bars depict the group medians and quartile ranges (Med (Q25; Q75). In Figure 3, the statistical analysis was performed with the Mann–Whitney U test.

To examine the absolute numbers and percentages of major T cell subsets in acute and convalescent COVID-19 individuals, we analyzed CD3, CD4, and CD8 co-expression in three groups of patients by using flow cytometry (Figure 3, Supplementary Table S1). Both the percentage and absolute number of CD3+ T cells (Figure 3A,D) were shown to decrease in patients with acute COVID-19 compared to healthy controls (74.17% (65.54; 79.45) vs. 78.49% (73.81; 80.77) with *p* = 0.010 and 855 (562; 1139) cells per 1 μL (hereinafter) vs. 1270 (1090; 1580) cells per 1 μL with *p* < 0.001, respectively). Next, the CD4+ T cell level in peripheral blood samples (Figure 1B,E) from patients with acute COVID-19 was lower than in COVID-19 convalescents and healthy controls (527 (399; 743) cells per 1 μL vs. 866 (703; 960) cells per 1 μL and 775 (671; 1053) cells per 1 μL with *p* < 0.001 in both groups, respectively). Finally, patients with acute COVID-19 showed decreased relative numbers of CD8+ T cells (Figure 3C,F) compared to the control (22.70% (18.23; 29.65) vs. 24.96% (21.00; 29.56), *p* = 0.010) and altered absolute numbers of circulating CD3+CD8+ cells compared to COVID-19 convalescents and healthy controls (285 (179; 382) cells vs. 520 (355; 600) and 444 (351; 525) cells with *p* < 0.001 in both cases, respectively).

**Figure 4:**
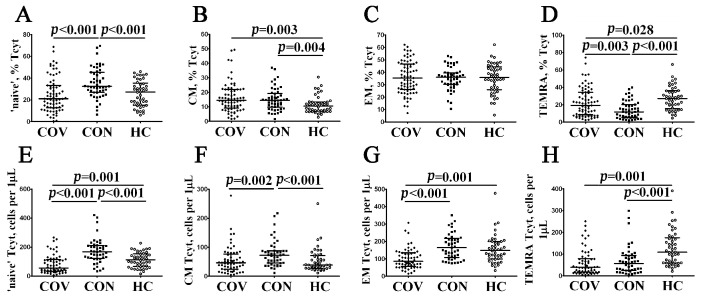
Alteration in relative and absolute numbers of major CD8+ T cell subsets with varying patterns of CD45RA and CD62L expression in acute and convalescent COVID-19 patients. Scatter plots (**A**–**D**) and (**E**–**H**) show the percentages and absolute numbers of ‘naïve’ (CD45RA+CD62L+), central memory (CM, CD45RA−CD62L+), effector memory (EM, CD45RA−CD62L−), and terminally differentiated CD45RA-positive effector memory (TEMRA, CD45RA+CD62L−) CD8+ T cells, respectively. Black circles denote patients with acute COVID-19 (COV, *n =* 71); black squares—convalescent COVID-19 individuals (CON, *n =* 51); white circles—healthy control (HC, *n =* 46). Each dot represents individual subjects, and horizontal bars depict the group medians and quartile ranges (Med (Q25; Q75)). In Figure 4, the statistical analysis was performed with the Mann–Whitney U test.

Next, using multicolor flow cytometry, we assessed the percentage and relative numbers of circulating CD8+ T cell subsets classified by CD45RA and CD62L co-expression. This approach, which was based on the expression of the leukocyte common antigen isoform CD45RA and the cell adhesion molecule CD62L, divided CD8+ T cells into naïve (CD45RA+CD62L+), central memory (CM, CD45RA–CD62L+), effector memory (EM, CD45RA–CD62L−), and terminally differentiated CD45RA-positive effector memory (TEMRA, CD45RA+CD62L−) cells [28]. The data obtained are summarized in Figure 4 and Supplementary Table S2. We found that the relative number of CM CD8+ T cells was increased, whereas the level of TEMRA CD8+ T cells was decreased in patients with acute COVID-19 compared to healthy controls (14.46% (9.18; 21.97) vs. 10.48% (6.98; 13.22) with *p* = 0.003 and 19.24% (8.43; 34.45) vs. 27.10% (16.21; 35.92) with *p* = 0.028, respectively), but the level of all mature CD8+ T cells was significantly lower in acute COVID-19 in comparison with convalescent-phase and healthy individuals (except CM CD8+ T cells, Figure 4). Interestingly, we found that COVID-19 convalescent patients showed increased percentages and absolute numbers of circulating ‘naïve’ (32.53% (26.84; 45.47) vs. 27.22% (15.62; 34.92) and 167 (125; 209) cells vs. 112 (68; 150) cells with *p* < 0.001 in both cases, respectively) and CM (14.49% (9.81; 19.11) vs. 10.48% (6.98; 13.22), *p* = 0.004 and 72 (46; 87) cells per 1 μL vs. 39 (28; 68) cells per 1 μL, *p* < 0.001) CD8+ T cells, whereas the frequency of TEMRA CD8+ T cells was decreased compared to healthy volunteers (11.79% (5.39; 20.24) vs. 27.10% (16.21; 35.92), *p* < 0.001 and 57 (25; 93) cells vs. 109 (60; 170) cells, *p* < 0.001).

### 3.2. Imbalance in EM and TEMRA CD8+ T Cell Subsets in Acute and Convalescent COVID-19 Patients

**Figure 5:**
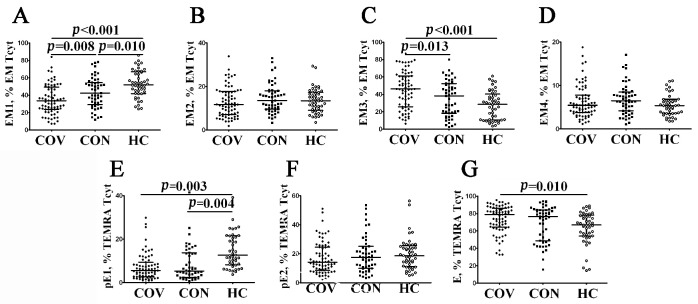
Alterations in relative number of EM and TEMRA CD8+ T cell subsets with different patterns of CD27 and CD28 expression in acute and convalescent COVID-19 patients w. Scatter plots (**A**–**D**)—EM CD8+ T cell were subdivided into EM1 (CD27+CD28+), EM2 (CD27+CD28−), EM3 (CD27−CD28−), and EM4 (CD27−CD28+) subsets, respectively. Scatter plots (**E**–**G**)—TEMRA CD8+ T cells were subdivided into CD27+CD28+ pE1, CD27+CD28− pE2, and CD27–CD28– E subsets, respectively. Black circles denote patients with acute COVID-19 (COV, *n =* 71); black squares—convalescent COVID-19 individuals (CON, *n =* 51); white circles—healthy control (HC, *n =* 46). Each dot represents individual subjects, and horizontal bars depict the group medians and quartile ranges (Med (Q25; Q75)). In Figure 5, the statistical analysis was performed with the Mann–Whitney U test.

Because patients with acute COVID-19 had an altered distribution of major peripheral blood CD8+ T cell subsets, we investigated changes in EM and TEMRA CD8+ T cell populations. It is well known that the expression of CD27 and CD28 co-stimulatory molecules allows one to define the four distinct effector memory subsets [29]. Thus, a multicolor flow cytometric analysis resulted in identifying EM1 (CD27+CD28+), EM2 (CD27+CD28−), EM3 (CD27−CD28−), and EM4 (CD27−CD28+) subsets (Figure 5 and Supplementary Table S3). The assessment of the CD8+ T cell maturation and differentiation stages revealed a significantly higher increase in the relative number of EM3 and decreased EM1 cytotoxic T cells in acute COVID-19 patients compared to COVID-19 convalescents (46.18% (25.76; 61.25) vs. 38.16% (18.35; 52.37), *p* = 0.013 and 33.53% (23.65; 49.34) vs. 42.43% (25.76; 61.25) with *p* = 0.008, respectively) and healthy individuals (46.18% (25.76; 61.25) vs. 28.67% (10.74; 39.05), *p* < 0.001 and 33.53% (23.65; 49.34) vs. 52.10% (42.05; 66.44) with *p* < 0.001, respectively). Of note, EM1 CD8+ T cells persisted at lower levels several weeks after recovery (Figure 5A). 

Next, the differentiation of terminally differentiated CD8+ T cells (TEMRA) by assessing CD27 and CD28 expression [30] allowed us to subdivide CD8+ T cells into pre-effector type 1 cells (pE1, CD27+CD28+), pre-effector type 2 cells (pE2, CD27+CD28–), and effector cells (E, CD27−CD28−). We noticed (Figure 5E–G, and Supplementary Table S3) that the most immature TEMRA CD8+ T cell subset—pE1—was significantly decreased in peripheral blood samples from acute and convalescent COVID-19 patients compared to healthy controls (5.53% (2.70; 9.38) and 5.35% (2.34; 13.77), respectively, vs. 12.60% (8.36; 21.53), *p* < 0.001 in both cases). Furthermore, the frequency of mature CD27–CD28– effector CD8+ T cells was increased during the acute phase of SARS-CoV-2 infection in comparison with the control group (78.80% (64.22; 86.16) vs. 67.03% (54.39; 77.54), *p* = 0.002).

### 3.3. Alterations in CD57 and Chemokine Receptor Expression on CD8+ T Cell Subsets from COVID-19 Patients and COVID-19 Convalescents

We first analyzed CD57 expression on CD8+ T cell subsets at different maturation stages (Table 1). Previously, it was demonstrated that CD57 expression was closely linked to the accumulation of major effector cytolytic molecules in granules, including granzyme A, granzyme B, and perforin [31]. We found that several CD8+ T cell subsets from patients with acute COVID-19, including ‘naïve’, central memory cells, and effector memory cells (along with EM1 and EM4), as well as pE1 and E cells within TEMRA subsets, had significantly increased surface CD57 level (Table 1). Furthermore, it was noted that CD57 expression on CD8+ T cells from COVID-19 convalescents was also altered, because we found increased levels of CD57 on ‘naïve’, CM, EM4, and pE1 CD8+ T cell subsets.

**Figure 6:**
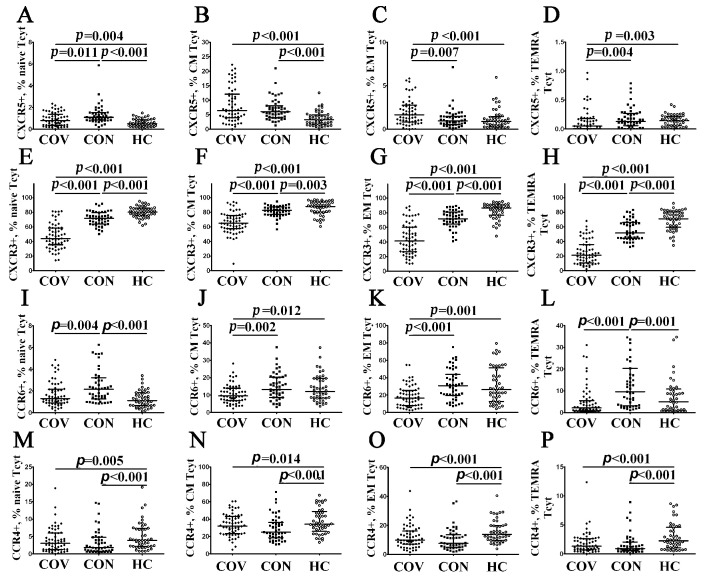
Chemokine receptor profiles for major CD8+ T cell subsets with varying patterns of CD45RA and CD62L expression in acute and convalescent COVID-19 patients. Scatter plots (**A**–**D**), (**E**–**H**), (**I**–**L**) and (**M**–**P**) show the percentages of ‘naïve’ (CD45RA+CD62L+), central memory (CM, CD45RA−CD62L+), effector memory (EM, CD45RA−CD62L−), and terminally differentiated CD45RA-positive effector memory (TEMRA, CD45RA+CD62L−) CD8+ T cells, respectively, expressing CXCR5, CXCR3, CCR6, and CCR4, respectively. Black circles denote patients with acute COVID-19 (COV, *n =* 71); black squares—convalescent COVID-19 individuals (CON, *n =* 51); white circles—healthy control (HC, *n =* 46). Each dot represents individual subjects, and horizontal bars depict the group medians and quartile ranges (Med (Q25; Q75). In Figure 6, the statistical analysis was performed with the Mann–Whitney U test.

Next, we investigated cell-surface CXCR5 expression allowing T cells to migrate from the lymph node T cell zone into B cell follicles, which were enriched with CXCL13 [32]. We found that the vast majority of CXCR5-expressing CD8+ T cells exhibited CM and EM phenotypes, whereas their level within ‘naïve’ and TEMRA subsets CD8+ T cell was less than 1% (Figure 6A–D, Supplementary Table S4). However, we found that the patients with acute COVID-19 had an increased level of CXCR5+ cell within CM and EM compared with healthy controls (6.35% (4.00; 11.83) vs. 3.22% (1.88; 4.67) and 1.60% (0.91; 2.69) vs. 0.85% (0.34; 1.37), *p* < 0.001 in both cases, respectively). Similarly, CM CD8+ T cells in COVID-19 convalescents also contained a higher level of CXCR5-expressing cells as compared with control group (6.07% (4.29; 8.76) vs. 3.22% (1.88; 4.67), *p* < 0.001). 

Further, we revealed CXCR3-expressing cells within the major CD8+ T cell maturation stages. It is known that CXCR3 facilitates the migration of T cells to inflamed tissue sites along a gradient of chemokines such as CXCL9, CXCL10, and CXCL11 [33,34]. Interestingly, we revealed dramatically decreased CXCR3 expression in all CD8+ T cell maturation subsets from patients with acute COVID-19 vs. COVID-19 convalescents and healthy controls (Figure 6E–H, Supplementary Table S4). Similarly, COVID-19 convalescents also exhibited low levels of CXCR3+ cells within ‘naïve’, CM, EM, and TEMRA CD8+ T cells. 

Next, we identified CD8+ T cells that expressed CCR6 necessary for migration to mucosal tissues enriched with CCL20 [35]. We found that CCR6-positive CD8+ T cells were present within CM, EM, and TEMRA subsets (Figure 6J,K, Supplementary Table S4). Of note, circulating CM and EM CCR6+CD8+ T cells were decreased in patients with acute COVID-19 compared to COVID-19 convalescents (9.49% (6.71; 13.85) vs. 13.91% (8.70; 20.15) with *p* = 0.002 and 17.11% (8.39; 25.39) vs. 29.79% (19.09; 43.85), *p* < 0.001, respectively) and healthy volunteers (9.49% (6.71; 13.85) vs. 12.73% (8.22; 19.58) with *p* = 0.012 and 17.11% (8.39; 25.39) vs.26.35% (12.84; 47.73), *p* = 0.001, respectively). Nevertheless, an increased number of CCR6+ cells was noticed in ‘naïve’ and TEMRA CD8+ T cell subsets from COVID-19 convalescents compared to healthy controls (1.76% (1.26; 2.96) vs. 1.16% (0.69; 1.89) and 11.40% (3.27; 20.37) vs. 4.99% (1.39; 10.15), respectively, *p* < 0.001 in both cases). 

Finally, we defined CD8+ T cells that expressed the chemokine receptor CCR4, which may interact with CCL17 and CCL22, as being critical for skin homing [36]. Interestingly, we revealed that patients with acute COVID-19, as well as COVID-19 convalescents, showed decreased frequencies of CCR4-expressing cells within all CD8+ T cell maturation subsets compared to healthy controls (Figure 6M–P, Supplementary Table S4). 

### 3.4. Imbalance in Peripheral Blood Tc1, Tc2 and Tc17 Cells from COVID-19 Patients and Convalescents

To assess relevant ‘polarized’ CD8+ T cell subsets, we studied the peripheral blood cells by multi-color staining for a set of the following chemokine receptors: CXCR5, CXCR3, CCR4, and CCR6, as proposed earlier [37,38]. Thus, we identified Tc1 (CCR6–CXCR3+), Tc2 (CCR6–CXCR3–), Tc17 (CCR6+CXCR3–), and double-positive Tc17.1 (CCR6+CXCR3+) in our blood samples.

**Figure 7:**
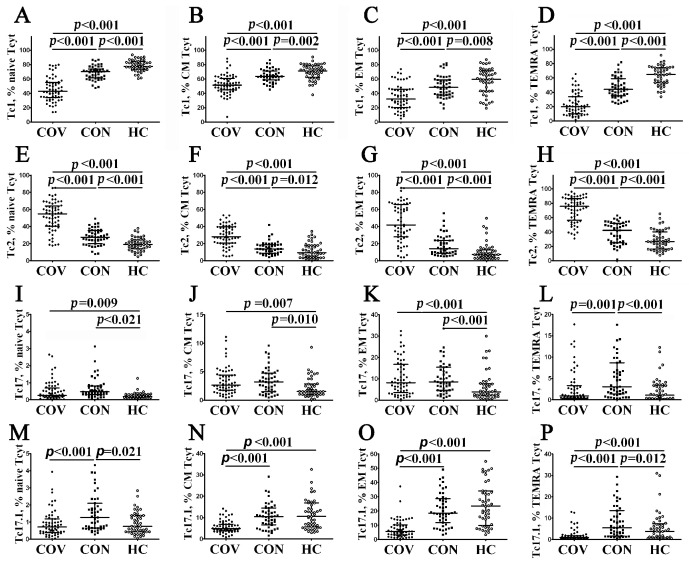
Imbalance in peripheral blood Tc1, Tc2, Tc17, and Tc17.1 cells in major CD8+ T cell subsets with varying patterns of CD45RA and CD62L expression in acute and convalescent COVID-19 patients. Scatter plots (**A**–**D**), (**E**–**H**), (**I**–**L**), and (**M**–**P**) show the relative numbers of Tc1 (CCR6−CXCR3+), Tc2 (CCR6−CXCR3−), Tc17 (CCR6+CXCR3−), and double-positive Tc17.1 (CCR6+CXCR3+) cells within ‘naïve’ (CD45RA+CCR7+), central memory (CM, CD45RA−CCR7+), effector memory (EM, CD45RA−CCR7−), and terminally differentiated CD45RA-positive effector memory (TEMRA, CD45RA+CCR7−) CD8+ T cells, respectively. Black circles denote patients with acute COVID-19 (COV, *n =* 71); black squares—convalescent COVID-19 individuals (CON, *n =* 51); white circles—healthy control (HC, *n =* 46). Each dot represents individual subjects, and horizontal bars depict the group medians and quartile ranges (Med (Q25; Q75)). In Figure 7, the statistical analysis was performed with the Mann–Whitney U test.

Thus, we first analyzed the frequency of CCR6–CXCR3+ Tc1 within ‘naïve’, CM, EM, and TEMRA CD8+ T cells in patients with acute COVID-19 (Figure 7A–D) and found that the level of Tc1 cell in all CD8+ T cell subsets decreased compared to convalescent COVID-19 individuals and healthy controls. Furthermore, the levels of ‘naïve’, CM, EM, and TEMRA Tc1 cells were lower in COVID-19 convalescents than in the control group (Figure 7A–D, Supplementary Table S5). Next, compared with the other, the percentage of CCR6–CXCR3– Tc2 cells peaked in patients with acute COVID-19 (Figure 7E–H, Supplementary Table S5). Moreover, COVID-19 convalescents also had increased Tc2 frequencies compared to healthy controls. Similarly to CCR6 expression, CCR6+CXCR3– Tc17 cells were primarily identified within CM and EM CD8+ T cell subsets, and the percentage of Tc17 cells was higher during acute SARS-CoV-2 infection, as well as in COVID-19 convalescents, than in healthy controls (2.63% (1.73; 4.36) and 3.16% (1.50; 4.73) vs. 1.66% (1.03; 3.30) with *p* = 0.007 and *p* = 0.010, respectively, for CM subsets, and 8.13% (4.15; 16.75) and 8.46% (4.64; 14.27) vs. 3.86% (2.23; 7.51), *p* < 0.001 in both cases for the EM CD8+ T cell subset). However, the levels of CM and EM Tc17 cells were similar in both acute and convalescent COVID-19 patients (Figure 7I–L, Supplementary Table S5). Finally, we found that CM and EM Tc17.1 (CCR6+CXCR3+) were dramatically decreased in patients with acute COVID-19 compared to COVID-19 convalescents and healthy controls (4.88% (3.73; 7.25) vs. 10.52% (6.64; 13.12), *p* < 0.001 and 11.09% (5.80; 16.87), *p* < 0.001 and 5.69% (3.23; 10.77) vs. 18.47% (11.92; 28.95), *p* < 0.001 and 23.53% (9.94; 33.59), *p* < 0.001) (Figure 7M–P).

### 3.5. Elevated Serum IL-27 Level Negatively Correlates with CCR6+CD8+ T Cell Subsets in Patients with Acute COVID-19

**Figure 8:**
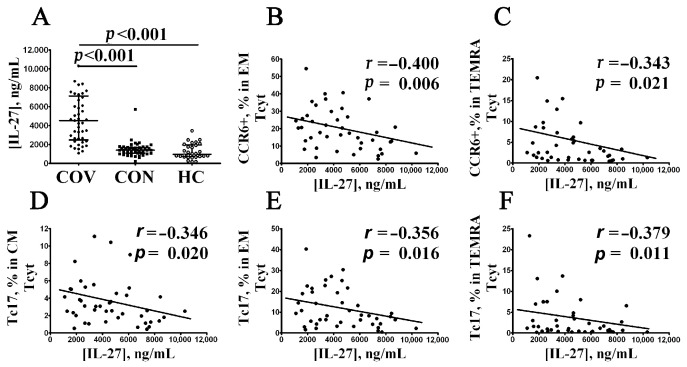
Correlation between elevated serum IL-27 level and CCR6+CD8+ T cells in acute COVID-19 patients. Comments: (**A**) IL-27 concentration (ng/mL) in patients with acute COVID-19 (black circles, COV, *n =* 45); convalescent COVID-19 individuals (black squares, CON, *n =* 48), and healthy control (white circles, HC, *n =* 30). Each dot represents individual subjects, and horizontal bars depict the group medians and quartile ranges (Med (Q25; Q75)). (**B**–**F**) Spearman correlation coefficient between serum IL-27 concentration and level of peripheral blood CCR6+ EM and CCR6+ TEMRA CD8+ T cells, as well as Tc17 CD8+ T cell subsets within CM, EM, and TEMRA CD8+ T cells, respectively. In Figure 8, the statistical analysis was performed with the Mann–Whitney U test.

Previously, we found increased levels of 18 cytokines (including IL-6, IL-7, IL-15, IL-27, TNFα, TNFβ, CCL2/MCP-1, CCL7/MCP-3, CXCL1/GROα, CXCL8/IL-8, CXCL10/IP-10, CXCL9/MIG, IL-1ra, IL-10, M-CSF, GM-CSF, and VEGF-A) in blood plasma from COVID-19 patients the during the acute phase of the SARS-CoV-2 infection vs. healthy controls [39]. Accordingly, we used a correlation analysis to examine the relationship between the levels for all aforementioned peripheral blood ‘polarized’ CD8+ T cell subsets and serum cytokine profiles in patients with acute COVID-19, COVID-19 convalescents, and healthy volunteers. Surprisingly, it was observed that only IL-27 levels negatively correlated with several CCR6-positive CD8+ T cell subsets (Figure 8A). At the beginning, there was an increased serum IL-27 level noted in patients with acute COVID-19 compared to COVID-19 convalescents and healthy controls (4533 ng/mL (2494; 7144) vs. 1422 ng/mL (1081; 1699) and 966 ng/mL (733; 1944), respectively, *p* < 0.001 in both cases). Next, we found that IL-27 negatively correlated with the CCR6+CD8+ T cell frequencies within EM (r = –0.400, *p* = 0.006) and TEMRA (r = –0.343, *p* = 0.021) subsets in patients with acute COVID-19, but not in convalescents COVID-19 or healthy controls (Figure 8B,C, respectively). In addition, serum IL-27 levels also negatively correlated with Tc17 cells’ frequency within CM (r = –0.346, *p* = 0.020), EM (r = –0.356, *p* = 0.016), and TEMRA (r = –0.378, *p* = 0.011) CD8+ T cells from patients with acute COVID-19 (Figure 8D–F, respectively), but not in COVID-19 convalescents and healthy controls.

## 4. Discussion

Primarily, we found that patients with acute COVID-19 showed decreased absolute numbers of CD3+CD8+ cells compared to healthy controls and COVID-19 convalescents. Similarly, several studies, including Mann et al. [40], Mathew et al. [41], and Gao et al. [42], demonstrated that COVID–19 patients vs. the control group had lowered peripheral blood T cell count that correlated with deteriorated disease severity. Moreover, peripheral blood T cell counts were inversely correlated with serum IL-6 and IL-10 levels, which increased along with disease severity deterioration [43]. Moreover, a relation between blood T cell count and COVID-19 severity based on APACHE III score was uncovered [6]. It was found that the absolute peripheral blood CD8+ count in acute COVID-19 was decreased [44,45,46]. Several research groups suggested that COVID–19 patients with unfavorable vs. favorable outcomes were characterized by very low peripheral blood CD3+CD8+ T cell levels [47], which might be related to developing ARDS in acute COVID-19 [48]. Furthermore, plasma levels of CD8+ extracellular vesicles in patients with moderate COVID-19 were significantly decreased compared with healthy controls [49].

Moreover, we noticed that the CD3+CD8+ T cell subsets undergoing diverse maturation stages were markedly altered (summarized in Table 2). We found that the frequency of circulating CM and TEMRA CD8+ T cells in acute COVID-19 vs. HC was elevated. For instance, the first study by Mann et al. assessing the impact of SARS-CoV-2 infection vs. healthy volunteers on CD8+ T cell subset composition noted a decline in peripheral blood-naïve CD3+CD8+ T cells [40]. In particular, COVID-19 patients were characterized by a decreased percentage of CD45RA−CD27+CCR7− EM1 cells, whereas the proportion of EM2 and EMRA CD3+CD8+ T cells (bearing phenotype CD45RA−CD27−CCR7+ and CD45RA+CD27−CCR7−, respectively) at the onset of SARS-CoV-2 infection was significantly elevated compared with the control group [41]. Similar data were also obtained by De Biasi et al. revealing a reduced proportion of naïve as well as central memory CCR7+CD45RA+CD28+CD27+ and CCR7−CD45RA+CD28+CD27+/−, respectively, CD3+CD8+ T cells [44]. Moreover, Odak et al. [50] and Kratzer et al. [51] reported that the level of effector memory CD45RO+CCR7– CD8+ T cells in acute COVID-19 was substantially elevated compared with the control group. In addition, it was also shown that mild COVID-19 was parallel to an increased percentage of TEMRA CD8+ T cells along with a reduced naive and EM T cell proportion, whereas severe COVID-19 was associated with a decline in naïve, but elevated EM CD8+ T cells [52]. Whereas severe COVID-19 vs. the control group featured a high percentage of CD45RO+CD45RA– CD8+ T cells, further analysis revealed that such a disease course was also associated with a high count of effector memory CD8+ T cells (CD27−CCR7−) that was parallel to a low level of transitional memory CD27+CCR7– CD8+ T cells [19]. Moreover, patients requiring mechanical ventilation vs. mild COVID-19 were noted to have lower percentages of CD45RA–CCR7– CD8+ T cells along with elevated levels of CD45RA+CCR7– TEMRA CD8+ T cells [45].

A detailed analysis of EM and TEMRA CD8+ T cells in acute COVID-19 allowed us to demonstrate that effector T cell subsets (CD27–CD28– EM3 within total EM subsets and CD27–CD28– effector cells within the total TEMRA subset) were elevated along with decreased levels of EM1 and pE1 cells during the acute phase of SARS-CoV-2 infection. Interestingly, EM1 and pE1 CD8+ T cells showed high proliferative potential but were unable to display effector properties, while CD27–CD28– EM1 cells and effector cells had high cytotoxic activity [29,30]. Similar data were obtained by Ramljak et al. revealing that mild COVID-19 was associated with a decreased proportion of EM1 (CD28+CD27+) and EM4 (CD28+CD27−) subsets within peripheral blood CD45RA–CCR7– EM CD8+ T cells compared with the healthy control [46]. Moreover, we also uncovered that decreased levels of EM1 and pE1 CD8+ T cells were sustained long term after the acute stage of COVID-19, thereby pointing to the need for prolonged monitoring of EM and TEMRA subset recovery.

Thus, we consider that alteration in CD8+ T cell subset composition could be related to both the efficient generation of the effector cell subset and impaired differentiation of cytotoxic T cells in the thymus. The latter was confirmed by both decreased levels of recent thymic emigrant CD3+CD8+CD45RA+CD62L+CD31+ T cells [51] as well as TRECs [53,54]. In addition, this might be due to the disturbed structure of the secondary lymphoid organs enabled in mediating antigen-dependent CD8+ T cell differentiation, which is in line with previous studies [22,23,24]. Moreover, recent data emphasize an altered balance between diverse helper T cell subsets [55,56,57] as well as lowered Th1 cell functional activity and an altered phenotype thereof [58,59,60], which might also affect CD8+ T cell maturation, differentiation, and polarization.

Our study demonstrated that several months after the onset of acute SARS-CoV-2 infection, peripheral blood samples contained elevated percentages and absolute numbers of ‘naïve’ and central memory CD8+ T cells, whereas the level of TEMRA cells was decreased compared with the control group. Rajamanickam et al. revealed that the levels of central and effector memory CD8+ T cells, as well as terminal effector (bearing the phenotypes CD45RA–CCR7+CD95+CD28+, CD45RA–CCR7–CD95+CD28, and CD45RA–CCR7–CD95+CD28–, respectively) cells, were elevated within 15–30 to 61–90 days PSO that was paralleled with a simultaneous decline in ‘naïve’ and transitional memory (CD45RA+CCR7+CD95–CD28+ and CD45RA+CCR7–CD95+CD28+, respectively) subsets so that all cell populations reached a plateau [61]. On the other hand, COVID-19 convalescents vs. control subjects were found to sustain elevated levels of effector memory CD3+CD8+CD45RO+CCR7– CD8+ T cells [51]. Recently, Wiech et al. demonstrated that CD8+ T cells from convalescent patients exhibited a low proportion of naïve cell population at 6 months PSO [62]. Taken together, our results, as well as literary data, point to alterations in CD8+ T cell maturation in patients at extended time points after acute SARS-CoV-2 infection.

We found that the frequency of CD8+ T cells expressing CD57 was increased in patients with acute COVID-19 and convalescents (Table 2). There are also publications showing that the percentage of mature perforin-positive CD3+CD8+ T cells increased, which was further closely related to deteriorated disease severity as well as increased serum CRP levels in COVID-19 patients [41]. An increasing proportion of CD57+CD8+ T cells was noted in other reports as well [45]. However, in response to in vitro stimulation, CD8+ T cells downmodulated CD107a expression compared to control subjects [63]. On the other hand, it was evidenced that the quantity of lung parenchymal CD8+ T cells was not prominently increased during severe COVID-19 compared to the control group, albeit the majority of such cells expressed the PD-1 molecule, but they virtually lacked intracellular granzyme B expression [64]. Moreover, we also identified an increased presence of senescent CD57-expressing cells within ‘naïve’, CM, and EM CD8+ T cells after several months of recovery. Our data are in agreement with a report in which elevated CD57+CD8+ T cells were found in circulation up to 6 months PSO [62]. Moreover, CD57 is a key marker of immune cell senescence, and its increased expression could be associated with prolonged chronic infection [41,44]. Furthermore, immunosenescence could include a shift towards less functional CD8+ T cells of different subsets, including ‘naïve’ and memory cells. Taken together, our results and literary data point to altered phenotype and functional activity of ‘naïve’ and memory CD8+ T cells in patients with acute COVID-19 and COVID-19 convalescents. Additionally, it is worth noting that activated CD38+HLA-DR+CD8+ T cells from acute COVID-19 patients contained higher levels of intracellular granzyme A and B along with perforin proteins vs. convalescent COVID-19 patients or control subjects [65]. In the case of cytotoxic CD8+ T cells, it was noted that they upregulated PD-1 and TIM-3 expression, recognized as cell senescence markers [55]. Moreover, such data were also closely related to the progression of COVID-19 because, in severe vs. mild disease courses, the level of such peripheral blood T cells was elevated [66]. In addition, peripheral blood CD3+CD8+ cells also upregulated expression of other inhibitory surface markers such as BTLA and TIGIT, which may be referred to as molecules negatively regulating T cell effector properties [58]. Patients with severe vs. mild COVID-19 or control subjects were noted to have elevated levels of peripheral blood PD-1+ and CTLA-4+ cytotoxic T cells [52]. Recently, the list of surface checkpoint inhibitors, whose levels become sharply elevated on diverse T cell subsets including CD8+ T cells, has been extended including VISTA, LAG-3, CD160, NKG2A, Galectin-9, Galectin-3, PD-L1, PD-L2, LSECtin, and CD112 [67]. The expression of exhaustion markers PD-1 and TIM-3 were elevated on CD8+ T cells at month 8 PSO [68]. Thus, CD8+ T cells showed exhausting phenotypes in convalescent patients after acute SARS-CoV-2 infection for many months PSO. Such prolonged remodeling of immune response might take part in forming symptoms associated with post-COVID [69,70].

Apart from impaired events in CD8+ T cell differentiation and associated upregulated expression of cell senescence markers, profound changes also occur in homing molecule levels as was shown by us and others (Table 2). In particular, severe COVID-19 patients linked to pulmonary failure and requiring mechanical ventilation were evidenced to have upregulated cytotoxic T cell CCR4 and CCR5 levels, responsible for migration to lung tissue and promoting recruitment to the site of inflammation, respectively, whereas the percentage of CCR7+ cells was decreased compared not only to control subjects but also patients with mild COVID-19 [52]. Upon that, CCR6 expression was downmodulated on CD8+ T cells both in mild and severe disease courses, whereas CXCR3 levels remained virtually intact in all patient groups. Furthermore, Liechti et al. showed that ‘naïve’ and transitional memory CD8+ T cells from patients with severe and critical COVID-19 expressed higher levels of CCR4 compared to patients with moderate COVID-1; thus, enhanced homing of CD8+ T cells to the lung tissue could be linked to COVID-19 severity [71]. Georg et al. demonstrated that acute COVID-19 vs. the control group was associated with high surface CCR6 and CXCR3 levels on CD8+ T cells [72]. Interestingly, the total number of CD8+ T cells co-expressing CD69 and CXCR4 (necessary for migration to inflamed lung tissue) was found in peripheral blood and BAL fluid from COVID-19 patients, whereas long-term detection of this cell type in the circulation was tightly linked to unfavorable disease outcomes [19]. Altogether, these data suggest enhanced recruitment of effector immune cells to foci of inflammation along with the restrained potential for effector CD8+ T cells to return to the secondary lymphoid tissues. Elevated levels of CCR4+CD8+ T cells may contribute to a stronger immune response occurring within lung tissue. It may be presumed that CCR4-positive T cells migrate to pulmonary tissue resulting in respiratory distress and even respiratory failure in COVID-19 patients.

We also noted that the proportion of CCR6+CD8+ T cells in acute vs. convalescent COVID-19 patients was decreased, which was additionally revealed for CM and EM CD8+ T cell subsets compared with control subjects, which might be due to the migration of such cell types to peripheral inflamed host tissues (Table 2). Indeed, elevated levels of CCR6+CD8+ T cells were observed in the BAL fluid in acute COVID-19, which might be due to the chemokine CCL20 being overproduced by alveolar macrophages and other cell types at the site of inflammation [73]. However, functions of CCR6CD8+ T cells still remain poorly investigated, but it was shown that such a T cell subset exerts a weak cytolytic potential (the expression of intracellular granzyme A was parallel to a low level of granzyme B and perforin), but able to produce effector cytokines such as TNFα and IFNγ. Presumably, such cells might function in mucosal layers both in disease and health [74]. Interestingly, this same cell type is maintained at high levels even after the acute phase of COVID-19, especially within ‘naïve’ and TEMRA subsets, which could suggest extensive polarization of ‘naïve’ cells towards effector CCR6+CD8+ T cells. This might be due to prolonged maintenance of both SARS-CoV-2 particles and virus-derived fragments/antigens in mucosal membranes, which promotes a chronic course of the immune antiviral response [75,76].

Next, we also demonstrated that acute COVID-19 was noted to have upregulated CXCR5 expression on diverse cytotoxic CD8+ T cell subsets (Table 2). In particular, it was shown that a bronchoscopy of critically ill COVID-19 patients requiring invasive mechanical ventilation (IMV) detected a great quantity of activated CXCR5 co-expressing CD38+CD8+ T cells [77]. CXCR5-positive CD8+ T cells were found both in lymphoid follicles within human peripheral lymphoid organs and peripheral blood [78]. It is believed that, owing to CXCR5, this cell type is able to enter the B-cell zone in peripheral lymphoid organs and expresses surface co-stimulatory molecules CD70, OX40, and ICOS, seemingly allowing them to promote survival and elevated IgG secretion in in vitro B cell co-cultures. Upon that, CD8+CXCR5+ T cells expressed virtually no cytolytic molecules (perforin and granzyme B) but were positive for cytosolic granzyme A protein [78,79]. However, currently, CD8+CXCR5+ T cells are recognized as an extremely heterogenous lymphocyte population executing diverse functions, not being solely limited by regulating the humoral arm of the immune response [80,81]. The level of peripheral blood CXCR5+CD8+ T cells was elevated during the progression of both community-acquired and nosocomial pneumonia, which might also serve as a diagnostic marker to assess disease severity and identify subsequent exacerbations [82]. Adenoid tissues isolated from COVID-19 convalescents were noted to have an increased proportion of CD8+CXCR5+ T cells, additionally expressing markers CD69 and CD103 typical of tissue-resident memory cells [83]. Thus, in both groups of patients, we found an increased level of CXCR5+ cells within CM CD8+ T cells, which were able to migrate to B cell-dependent areas of peripheral lymphoid tissue and could affect CD19+ B cell functions. Interestingly, Tfh cells’ frequency in COVID-19 was lowered, regardless of COVID-19 severity [56], but within central memory Th cells, a lower level of CXCR5-expressing CD4+ T cells was found in patients with severe COVID-19 vs. patients with moderate COVID-19 [57]. However, some previous studies found elevated levels of peripheral blood memory Tfh cells [58] in patients with acute COVID-19 or no differences with healthy controls [6]. Furthermore, it was shown that COVID-19 convalescent patients had elevated Tfh cell frequencies compared with HC [84], but Gong et al. noted no differences in circulating follicular Th cell frequencies between patients that had recovered from COVID-19 and healthy individuals [85]. Thus, CXCR5+CD8+ T cells as well as Tfh cells could play an important part in the regulation of cellular and humoral immunity during the acute phase of infection and post-COVID [86,87].

Hence, a long-term increase in peripheral blood CCR6- and CXCR5-expressing CD8+ T cells in COVID-19 convalescents revealed in our study might be considered one of the signals associated with long-COVID alterations in cytotoxic T cell subset composition. The data obtained further extend previous findings about the alteration in CD8+ T cell differentiation and maturation coupled with the high expression of inhibitory receptors and cell senescence markers, as well as inhibitory checkpoint receptors [67,88,89].

It was noted above that multi-color flow cytometry allows one to distinguish, in cytotoxic T cells, not only diverse maturation stages but also reveal separate cell subsets within polarized CD8+ T cells, including Tc1, Tc2, Tc9, Tc17, and regulatory CD8+ T cells [90]. Previously, it was shown that CD8+ Tc1 cells able to produce IFNγ, similar to Tc2 (CD8+IL-4+) and Tc17 (CD8+IL-17A+) cells, were lowered in convalescent COVID-19 patients compared to control subjects [91]. On the other hand, the levels of memory CD8+CXCR3+CCR6+ and CD45RO+CXCR3−CCR6+CD8+ T cell subsets were elevated in mild vs. moderate-to-severe COVID-19 patients [59]. In our study, we noted a markedly elevated proportion of peripheral blood Tc2 cells (Table 2), which should, however, be interpreted with care, because Tc2 cells were identified as lacking CXCR3 and CCR6 expression according to the commonly accepted gating strategy [37,38]. Upon that, we noted a decreased percentage of CCR4-positive CD8+ T cells both in acute and convalescent COVID-19 patients. It should be emphasized that an increased Th2 cell level was typical in severe COVID-19 [57], whereas the peak percentage of CXCR3–CCR6− Th2 cells was found in patients with unfavorable disease outcomes [92]. Saris et al. demonstrated that CD8+CCR6+, rather than CD8+CXCR3+ or CD8+CCR4+ T cells were recruited to lung tissue in intensive care unit patients [73]. Hence, it allows us to highlight a pro-inflammatory role for Tc17 cells aggravating inflammation during SARS-CoV-2 infection. Therefore, regulating the CCR6+CD8+T cell subset is a crucial aspect of COVID-19, whereas IL-27 might act as a potential regulator thereof. Increasing the CXCR3+CCR6− CD8+ T cell (Tc1) level was noted in adenoid tissue from convalescent COVID-19 patients compared to control subjects [83]. Moreover, it was noted that in vitro stimulated CD8+ T cells were featured with lowered potential to produce the key cytokines such as IFNγ and IL-2 [45,63]. Despite this, De Biasi et al. demonstrated opposite data highlighting augmented effector properties in CD8+ T cells from COVID-19 patients such as elevated IL-2 and IL-17A in vitro production as well as higher expression of the CD107a degranulation marker compared with healthy subjects [44].

IL-27 is a heterodimeric molecule composed of two subunits—Epstein–Barr virus-induced gene 3 (EBI3) and IL-27p28. Its cognate receptor is presented by IL-27R consisting of the subunits gp130 and IL-27Ralpha [93], found in high levels on NK cells and T cells, so its quantity on human CD8+ T cells vs. CD4+ T cells is higher [94]. Acting via IL-27R on CD8+ T cells, IL-27 may enhance related cytolytic potential by stimulating granzyme B production and augmenting IFNγ secretion [94]. SARS-CoV-2 virus, via its own viral RNA, may be sensed by pattern-recognition receptors TLR3 and TLR7, which results in activated transcription factor IRF3 and its subsequent nuclear translocation [95]. Along with that, IL-27 is produced due to IRF3 activation, which, in turn, mediates the antiviral response and inhibits Tc17 by restraining functional potential [96]. It was noted above that Tc17 mainly exerts secretory activity and contains lowered perforin and granzyme levels [97], whereas, e.g., Tc1 predominantly displays cytolytic potential [37]. Our study demonstrates that the serum IL-27 level was elevated in acute vs. convalescent COVID-19 patients, which is in line with a previous study by Tamayo-Velasco et al. showing that IL-27 might serve as one of the unfavorable prognostic markers [98]. Negative correlations found between Tc17 subsets and serum IL-27 levels revealed in our study during acute COVID-19 may suggest suppressed cytokine-producing Tc17 potential and CD8+ T cell differentiation skewed towards Tc1 as the most crucial cell type in eliminating intracellular pathogens [99]. Moreover, Tc1, along with cytolytic activity, displays the potential to secrete IFNγ, exerting antiviral activity [100] and contributing to IL-27 secretion [93].

## 5. Conclusions

Thus, cytotoxic CD8+ T cells were shown to be critical for controlling SARS-CoV-2 infection during the acute phase of COVID-19 and altered CD8+ T cell subset composition, dramatically influencing an efficient antiviral immune response. Overall, continuing further comprehensive investigations on COVID-19-related CD8+ T cell phenotypic profiling, as well as subset composition, may facilitate not only investigation of the disease pathogenesis, but also provide deeper insight into the role that these cell types might play in diverse acute viral infections.

Our observations also described an abnormal CD8+ T cell profile in COVID-19 convalescents, which resulted in lower frequencies of effector CD8+ T cell subsets (TEMRA and Tc1), a higher senescent state (upregulated CD57 expression on ‘naïve’ and memory cell), and higher frequencies of CD8+ T cell subsets expressing lung tissue and mucosal tissue homing molecules (Tc2, Tc17, and Tc17.1). Moreover, COVID-19 convalescents might face long-lasting CD8+ T cell dysfunctions, resulting in a potentially weaker antiviral and anticancer immunity as well as autoimmunity. Thus, the long-term consequences of COVID-19 are still poorly investigated, as is the role of CD8+ T cells in post-COVID-19 syndrome, both of which require further, deeper investigation.

## Figures and Tables

**Table 1:** CD57 expressed on diverse CD8+ T cell subsets in acute COVID-19 and convalescent COVID-19 individuals. The quantitative data (% of CD57-expressing cells within CD8+ T cell subset) are presented as median and quartile ranges (Med (Q25; Q75).

	Acute COVID-19 (COV, *n =* 71)	Convalescent COVID-19 (CON, *n =* 51)	Healthy Control (HC, *n =* 46)	Significant Differences
**‘naïve’** CD8+ T cells	8.00 (2.07; 13.68)	3.41 (1.32; 7.07)	1.05 (0.43; 1.82)	*p*_1_ = 0.005 *p*_2_ < 0.001 *p*_3_ < 0.001
**CM** CD8+ T cells	12.72 (7.21; 26.31)	10.51 (5.99; 14.89)	2.86 (1.32; 5.41)	*p*_1_ = 0.058 *p*_2_ < 0.001 *p*_3_ < 0.001
**EM** CD8+ T cells	49.47 (29.30; 62.15)	39.14 (29.74; 53.68)	32.35 (13.42; 43.84)	*p*_1_ = 0.061 *p*_2_ < 0.001 *p*_3_ = 0.017
**EM1** CD8+ T cells	7.18 (4.62; 11.17)	7.10 (4.06; 10.69)	5.27 (3.58; 7.01)	*p*_1_ = 0.678 *p*_2_ = 0.009 *p*_3_ = 0.053
**EM2** CD8+ T cells	37.83 (29.93; 51.46)	42.27 (32.59; 50.00)	33.90 (21.13; 49.50)	*p*_1_ = 0.408 *p*_2_ = 0.299 *p*_3_ = 0.115
**EM3** CD8+ T cells	85.49 (73.98; 90.30)	84.27 (76.15; 89.79)	81.67 (66.20; 90.40)	*p*_1_ = 0.821 *p*_2_ = 0.130 *p*_3_ = 0.251
**EM4** CD8+ T cells	11.42 (5.27; 22.74)	10.25 (6.21; 16.49)	5.33 (1.93; 12.29)	*p*_1_ = 0.217 *p*_2_ < 0.000 *p*_3_ = 0.005
**TEMRA** CD8+ T cells	61.70 (42.22; 72.79)	65.03 (51.85; 73.08)	59.49 (46.32; 67.91)	*p*_1_ = 0.398 *p*_2_ = 0.393 *p*_3_ = 0.103
**pE1** CD8+ T cells	10.35 (4.58; 19.06)	18.65 (9.10; 23.81)	2.51 (1.36; 4.85)	*p*_1_ = 0.027 *p*_2_ < 0.001 *p*_3_ < 0.001
**pE2** CD8+ T cells	37.67 (23.79; 48.89)	33.33 (23.08; 48.01)	33.83 (23.61; 43.85)	*p*_1_ = 0.577 *p*_2_ = 0.438 *p*_3_ = 0.977
**Eff** CD8+ T cells	70.51 (59.22; 81.87)	77.06 (66.67; 87.07)	76.56 (69.55; 85.47)	*p*_1_ = 0.031 *p*_2_ = 0.044 *p*_3_ = 0.891

*p*_1_—statistical differences between acute COVID-19 and COVID-19 convalescent patient groups assessed by using nonparametric Mann–Whitney U test; *p*_2_—statistical differences between acute COVID-19 and healthy control groups assessed by using nonparametric Mann–Whitney U tests; *p*_3_—statistical differences between COVID-19 convalescent patient and healthy control groups assessed by using nonparametric Mann-Whitney U tests.

**Table 2:** Summary of alterations in peripheral blood CD8+ T cell subsets from acute and convalescent COVID-19 patients.

CD8+ T Cell Subset	Patients with Acute COVID-19	Convalescent COVID-19 Patients
Maturation:		
%	↑ CM and TEMRA	↑ Naïve and CM; ↓ TEMRA
#	↓ Naïve, EM and TEMRA	↑ Naïve and CM; ↓ EM and TEMRA
CD57 expression, %	↑ Naïve, CM and EM	↑ Naïve, CM and EM
Chemokine receptors,:		
CXCR5, %	↑ Naïve, CM and EM; ↓ TEMRA	↑ Naïve and CM
CXCR3, %	↓ Naïve, CM, EM and TEMRA	↓ Naïve, CM, EM and TEMRA
CCR6, %	↓ CM and EM	↑ Naïve and TEMRA
CCR4, %	↓ Naïve, CM, EM and TEMRA	↓ Naïve, CM, EM and TEMRA
‘Polarized’ subsets:		
Tc1, %	↓ Naïve, CM, EM and TEMRA	↓ Naïve, CM, EM and TEMRA
Tc2, %	↑ Naïve, CM, EM and TEMRA	↑ Naïve, CM, EM and TEMRA
Tc17, %	↑ Naïve, CM, EM and TEMRA	↑ Naïve, CM, EM and TEMRA
Tc17.1, %	↓ CM, EM and TEMRA	↑ Naïve and TEMRA

**Note**: ↑ and ↓—significant increase and significant decrease in CD8+ T cell subsets, respectively, in patients with acute COVID-19 or convalescent COVID-19 patients vs. healthy control group; % and #—relative and absolute numbers of CD8+ T cell subsets, respectively.

## Data Availability

Not applicable.

## Supplementary Materials

The following supporting information can be downloaded at: https://www.mdpi.com/article/10.3390/v14091906/s1, Table S1: Altered relative and absolute rate of major peripheral blood T cell subsets in acute and convalescent COVID-19 patients. Table S2: Altered relative and absolute number of major CD8+ T cell subsets varied in CD45RA and CD62L expression pattern in acute and convalescent COVID-19 patients., Table S3: Altered relative numbers of EM and TEMRA CD8+ T cell subsets varied in CD27 and CD28 expression pattern in acute and convalescent COVID-19 patients. Supplementary, Table S4: Chemokine receptor profiles on major CD8+ T cell subsets varied in CD45RA and CD62L expression pattern in acute and convalescent COVID-19 patients. Table S5: Imbalance in peripheral blood major maturation CD8+T cell subsets Tc1, Tc2, Tc17 and Tc17.1 cell in acute and convalescent COVID-19 patients.
