# Supplementary material for: Heterogenous CD8+ T Cell Maturation and ‘Polarization’ in Acute and Convalescent COVID-19 Patients
Source: Viruses. 2022 Aug 28;14(9):1906. doi: 10.3390/v14091906 (PMC9504186; doi:10.3390/v14091906)
Supplement: Supplementary file 1 [file viruses-14-01906-s001.zip › viruses-1825570-supplementary.pdf]

Supplementary File.

Supplementary Table S1. Altered relative and absolute rate of major peripheral blood T cell subsets in acute and convalescent COVID-19 patients.

T cell subset		Acute COVID-19 (COV, n = 71)	Convalescent COVID-19 (CON, n = 51)	Healthy control (HC, n = 46)	P value
T cells CD3+	%	74.17 (65.54; 79.45)	77.36 (70.70; 80.16)	78.49 (73.81; 80.77)	p = 0.173<br>p = 0.010<br>p = 0.218
	#	885 (562; 1139)	1345 (1199; 1588)	1270 (1090; 1580)	p < 0.001<br>p < 0.001<br>p = 0.573
Th cells CD3+CD4+	%	47.55 (40.53; 53.83)	46.94 (41.97; 51.83)	47.63 (43.82; 52.73)	p = 0.629<br>p = 0.684<br>p = 0.326
	#	527 (399; 743)	865 (703; 960)	775 (671; 1053)	p < 0.001<br>p < 0.001<br>p = 0.552
Tcyt cells CD3+CD8+	%	22.70 (18.23; 29.65)	26.69 (20.97; 31.68)	24.96 (21.00; 29.56)	p = 0.021<br>p = 0.137<br>p = 0.365
	#	285 (180; 382)	520 (355; 600)	444 (351; 525)	p < 0.001<br>p < 0.001<br>p = 0.175

Comments: % – the relative number of T cells within total lymphocyte population; # - absolute number of cells per 1 µL of whole peripheral blood. The data are shown as group median and quartile ranges (Med (Q25; Q75). Statistical analysis was performed by using the Mann–Whitney U test. Hereinafter, all symbols denote similar parameters in Supplementary Table S2.

Supplementary Table S2. Altered relative and absolute number of major CD8+ T cell subsets varied in CD45RA and CD62L expression pattern in acute and convalescent COVID-19 patients.

T cell subset		Acute COVID-19 (COV, n = 71)	Convalescent COVID-19 (CON, n = 51)	Healthy control (HC, n = 46)	P value
'Naïve' Tcyt	%	21.07 (12.81; 33.30)	32.53 (26.84; 45.47)	27.22 (15.62; 34.92)	p < 0.001<br>p = 0.477<br>p = 0.001
	#	56 (33; 113)	167 (125; 209)	112 (68; 150)	p < 0.001<br>p = 0.001<br>p < 0.001
Central memory Tcyt	%	14.46 (9.18; 21.97)	14.49 (9.81; 19.11)	10.48 (6.98; 13.22)	p = 0.858<br>p = 0.003<br>p = 0.004
	#	46 (24; 77)	72 (46; 87)	39 (28; 68)	p = 0.002<br>p = 0.934<br>p = 0.001
Effector memory Tcyt	%	35.36 (26.12; 46.58)	36.00 (30.24; 39.54)	35.71 (26.11; 43.61)	p = 0.842<br>p = 0.793<br>p = 0.879
	#	86 (57; 135)	164 (113; 214)	149 (101; 200)	p < 0.001

					p < 0.001<br>p = 0.302
TEMRA Tcyt	%	19.24 (8.43; 34.45)	11.79 (5.39; 20.24)	27.10 (16.21; 35.92)	p = 0.003<br>p = 0.028<br>p < 0.001
	#	39 (17; 79)	57 (25; 93)	109 (60; 170)	p = 0.297<br>p < 0.001<br>p < 0.001

Supplementary Table S3. Altered relative numbers of EM and TEMRA CD8+ T cell subsets varied in CD27 and CD28 expression pattern in acute and convalescent COVID-19 patients.

Tcyt subset:	Acute COVID-19 (COV, n = 71)	Convalescent COVID-19 (CON, n = 51)	Healthy control (HC, n = 46)	P value
**Effector memory Tcyt subsets:**				
**EM1,** CD27+CD28+	33.53 (23.65; 49.34)	42.43 (29.30; 54.47)	52.10 (42.05; 66.44)	p = 0.008<br>p < 0.001<br>p = 0.010
**EM2,** CD27+CD28–	11.79 (7.06; 17.71)	13.50 (9.65; 18.04)	13.43 (9.45; 17.28)	p = 0.172<br>p = 0.360<br>p = 0.665
**EM3,** CD27–CD28–	46.18 (25.76; 61.25)	38.16 (18.35; 52.37)	28.67 (10.74; 39.05)	p = 0.013<br>p < 0.001<br>p = 0.056
**EM4,** CD27–CD28+	5.45 (3.81; 7.78)	6.49 (4.20; 8.48)	5.38 (3.62; 6.83)	p = 0.256<br>p = 0.558<br>p = 0.086
**TEMRA Tcyt subsets:**				
**pE1,** CD27+CD28+	5.53 (2.70; 9.38)	5.35 (2.34; 13.77)	12.60 (8.36; 21.53)	p = 0.490<br>p < 0.001<br>p < 0.001
**pE2,** CD27+CD28–	14.18 (8.48; 24.66)	17.53 (9.85; 25.19)	18.68 (11.54; 25.09)	p = 0.252<br>p = 0.076<br>p = 0.598
**Effectors,** CD27–CD28–	78.80 (64.22; 86.16)	76.59 (48.71; 84.22)	67.03 (54.39; 77.55)	p = 0.346<br>p = 0.002<br>p = 0.100

Supplementary Table S4. Chemokine receptor profiles on major CD8+ T cell subsets varied in CD45RA and CD62L expression pattern in acute and convalescent COVID-19 patients.

Tcyt subset:	Acute COVID-19 (COV, n = 71)	Convalescent COVID-19 (CON, n = 51)	Healthy control (HC, n = 46)	P value
**'Naïve' Tcyt:**				
**CXCR5+**	0.80 (0.38; 1.28)	1.08 (0.83; 1.44)	0.53 (0.31; 0.84)	p = 0.004<br>p = 0.011<br>p < 0.001
**CXCR3+**	45.55 (35.96; 58.28)	71.89 (64.39; 79.41)	81.63 (76.56; 85.86)	p < 0.001<br>p < 0.001<br>p < 0.001
**CCR6+**	1.31 (0.91; 2.12)	1.76 (1.26; 2.96)	1.16 (0.69; 1.89)	p = 0.004<br>p = 0.194

				p < 0.001
**CCR4+**	3.05 (1.33; 6.07)	1.94 (0.79; 4.78)	4.18 (2.25; 10.19)	p = 0.106<br>p = 0.005<br>p < 0.001
**CM Tcyt:**				
**CXCR5+**	6.35 (4.00; 11.83)	6.07 (4.29; 8.76)	3.22 (1.88; 4.67)	p = 0.746<br>p < 0.001<br>p < 0.001
**CXCR3+**	66.47 (57.60; 76.26)	82.76 (77.84; 87.30)	88.29 (80.48; 92.84)	p < 0.001<br>p < 0.001<br>p = 0.003
**CCR6+**	9.49 (6.71; 13.85)	13.91 (8.70; 20.15)	12.73 (8.22; 19.58)	p = 0.002<br>p = 0.012<br>p = 0.680
**CCR4+**	31.14 (22.38; 42.89)	26.67 (19.32; 35.50)	37.62 (28.77; 52.25)	p = 0.067<br>p = 0.015<br>p < 0.001
**EM Tcyt:**				
**CXCR5+**	1.60 (0.91; 2.69)	1.07 (0.63; 1.54)	0.85 (0.34; 1.37)	p = 0.007<br>p < 0.001<br>p = 0.119
**CXCR3+**	43.05 (26.85; 60.62)	72.11 (66.37; 81.11)	86.67 (77.83; 90.62)	p < 0.001<br>p < 0.001<br>p < 0.001
**CCR6+**	17.11 (8.39; 25.39)	29.79 (19.09; 43.85)	26.35 (12.84; 47.73)	p < 0.001<br>p = 0.001<br>p = 0.656
**CCR4+**	9.71 (6.90; 16.06)	9.31 (5.34; 14.46)	15.39 (10.67; 26.78)	p = 0.351<br>p < 0.001<br>p < 0.001
**TEMRA Tcyt**				
**CXCR5+**	0.05 (0.01; 0.18)	0.16 (0.06; 0.29)	0.14 (0.06; 0.22)	p = 0.003<br>p = 0.004<br>p = 0.421
**CXCR3+**	21.24 (11.55; 35.57)	51.32 (44.52; 65.05)	71.28 (60.96; 79.82)	p < 0.001<br>p < 0.001<br>p < 0.001
**CCR6+**	2.50 (0.91; 6.01)	11.40 (3.27; 20.37)	4.99 (1.39; 10.15)	p < 0.001<br>p = 0.058<br>p = 0.001
**CCR4+**	1.33 (0.71; 2.62)	1.10 (0.61; 2.10)	2.61 (1.19; 6.39)	p = 0.377<br>p < 0.001<br>p < 0.001

Supplementary Table S5. Imbalance in peripheral blood major maturation CD8+T cell subsets Tc1, Tc2, Tc17 and Tc17.1 cell in acute and convalescent COVID-19 patients.

Tcyt subset:	Acute COVID-19 (COV. n = 71)	Convalescent COVID-19 (CON. n = 51)	Healthy control (HC, n = 46)	P value
**'Naïve' Tcyt:**				
**Tc1**	44.36 (34.79; 55.31)	70.05 (62.07; 76.17)	80.38 (74.14; 84.69)	p < 0.001

				p < 0.001<br>p < 0.001
**Tc2**	53.32 (40.57; 63.42)	27.52 (20.07; 34.53)	18.19 (13.95; 23.08)	p < 0.001<br>p < 0.001<br>p < 0.001
**Tc17**	0.26 (0.18; 0.70)	0.42 (0.27; 0.73)	0.19 (0.08; 0.33)	p = 0.068<br>p = 0.009<br>p < 0.001
**Tc17.1**	0.66 (0.38; 1.12)	1.03 (0.65; 1.79)	0.87 (0.44; 1.41)	p < 0.001<br>p = 0.149<br>p = 0.021
**CM Tcyt:**				
**Tc1**	51.84 (45.18; 62.80)	63.72 (59.88; 71.23)	71.74 (63.25; 78.14)	p < 0.001<br>p < 0.001<br>p = 0.002
**Tc2**	27.36 (20.17; 39.67)	13.53 (8.50; 18.59)	8.78 (4.20; 16.42)	p < 0.001<br>p < 0.001<br>p = 0.012
**Tc17**	2.63 (1.73; 4.36)	3.16 (1.50; 4.73)	1.66 (1.03; 3.30)	p = 0.677<br>p = 0.007<br>p = 0.010
**Tc17.1**	4.88 (3.73; 7.25)	10.52 (6.64; 13.12)	11.09 (5.80; 16.87)	p < 0.001<br>p < 0.001<br>p = 0.492
**EM Tcyt:**				
**Tc1**	35.37 (20.50; 46.20)	48.60 (37.79; 59.56)	60.36 (43.18; 71.37)	p < 0.001<br>p < 0.001<br>p = 0.008
**Tc2**	40.41 (27.71; 64.14)	13.73 (9.09; 23.83)	7.05 (3.89; 11.68)	p < 0.001<br>p < 0.001<br>p < 0.001
**Tc17**	8.13 (4.14; 16.75)	8.46 (4.64; 14.27)	3.86 (2.23; 7.51)	p = 0.871<br>p = 0.001<br>p < 0.001
**Tc17.1**	5.69 (3.23; 10.77)	18.47 (11.92; 28.95)	23.53 (9.94; 33.59)	p < 0.001<br>p < 0.001<br>p = 0.322
**TEMRA Tcyt:**				
**Tc1**	20.16 (10.68; 34.03)	43.38 (35.87; 55.95)	65.53 (53.76; 74.65)	p < 0.001<br>p < 0.001<br>p < 0.001
**Tc2**	75.80 (56.43; 86.20)	42.51 (27.15; 51.33)	26.14 (16.78; 36.30)	p < 0.001<br>p < 0.001<br>p < 0.001
**Tc17**	1.01 (0.43; 3.77)	3.98 (1.38; 8.66)	1.17 (0.25; 3.26)	p = 0.001<br>p = 0.289<br>p < 0.001
**Tc17.1**	0.98 (0.35; 1.80)	6.23 (1.89; 13.52)	3.73 (1.04; 6.87)	p < 0.001<br>p < 0.001<br>p = 0.012
